# The Characterization of Regulatory T-Cell Profiles in Alzheimer’s Disease and Multiple Sclerosis

**DOI:** 10.1038/s41598-019-45433-3

**Published:** 2019-06-19

**Authors:** Fausta Ciccocioppo, Paola Lanuti, Laura Pierdomenico, Pasquale Simeone, Giuseppina Bologna, Eva Ercolino, Fabio Buttari, Roberta Fantozzi, Astrid Thomas, Marco Onofrj, Diego Centonze, Sebastiano Miscia, Marco Marchisio

**Affiliations:** 1 Department of Medicine and Aging Sciences, University “G. D’Annunzio”, Chieti-Pescara, Italy; 2 Department of Neuroscience, Imaging and Clinical Sciences, University “G. D’Annunzio”, Chieti-Pescara, Italy; 3 Center on Aging Science and Translational Medicine (Ce.S.I.-Me.T.), University “G. D’Annunzio”, Chieti-Pescara, Italy; 4 Laboratory of Synaptic Immunopathology, Department of Systems Medicine, Tor Vergata University, Rome, Italy; 5 Unit of Neurology, IRCCS Neuromed, Pozzilli, (IS) Italy

**Keywords:** Cellular neuroscience, Neuroimmunology

## Abstract

Regulatory T Cells (Tregs) are a T-lymphocyte subset involved in the maintenance of immune peripheral tolerance. Despite evidence of the adaptive immune system’s role in Alzheimer’s Disease (AD), the involvement of Tregs is still not clear. We focused on the Flow-Cytometry analysis of the Treg frequencies and phenotypes in the AD. The aim of the study is to analyse similarities and differences in Tregs profile between Alzheimer’s Disease and Multiple Sclerosis. Regulatory T Cells (CD4+/CD25high/CD127low-neg) were identified using an innovative Flow Cytometry method and subtyped as Resting (analysed CD45RApos/CD25dim), Activated (CD45RAneg/CD25bright) and Secreting (CD45RAneg/CD25dim) cells. Our data demonstrate a significant decrease in the total and Resting Tregs in AD patients when compared to healthy subjects. The percentage of the results of the Resting Tregs were also reduced in MS patients together with a parallel frequency increase of Activated Tregs. Our data suggest that altered Treg phenotypes observed in both diseases could play a role in the impairment of the Treg-mediated immunological tolerance, recalling a possible link between the two pathologies. Given that this study was conducted on a restricted population, if confirmed by a further and enlarged study, the implications of the autoimmune mechanisms in AD pathophysiology could open new immunotherapeutic perspectives based on Treg modulation.

## Introduction

Alzheimer’s Disease (AD) is the most common kind of neurodegenerative dementia in the elderly population, characterized by both cognitive and behavioural dysfunctions^1,2^. The Alzheimer’s Disease neuropathology is characterized by the Amyloid beta (Ab) deposition, as amyloid plaques within some specific brain regions, as well as by microglial activation and local inflammatory responses. When the microglia fails to remove the Ab peptide, this first immune-related event is not decisive, therefore the microglial activation becomes chronic as does the related production of pro-inflammatory cytokines^3^. The consequent chronic neuro-inflammation status is associated with a toxic cascade, leading to neuronal death and resulting in neurodegeneration^4^. Accumulating evidence suggests a relationship among adaptive immunity, neuroinflammation, and neurodegenerative pathologies^5–7^. Adaptive immunity, also known as acquired immunity, encloses the humoral and the cell-mediated response. These events are both involved in the immune surveillance and in homeostasis balance^8,9^. Under neurodegenerative conditions, as in the AD or in Parkinson’s Disease (PD)^10,11^, T lymphocytes are recruited into specific brain regions^11,12^ and high frequencies of reactive T lymphocytes have been found in the bloodstream of these patients, suggesting their key role in the physiopathology of neurodegenerative disorders^13^. According to their phenotypes and functions, T cells are subtyped as CD8+ Cytotoxic Lymphocytes (CTL), CD4+ helper T lymphocytes (Th, subtyped as Th1 and Th2 subsets), CD4+ regulatory T lymphocytes (Tregs, including natural occurring Tregs and adaptively induced Tregs) and conventional T cells (effector T cells and memory T cells)^13–15^. In the central nervous system, the CD4+ T cells orchestrate both innate and adaptive immune responses, through a complex network of cellular interactions involved in the control of tissue homeostasis^13^. Various studies that have been published have underlined a multifaceted involvement of the CD4+ T cells in the physiopathology of the AD, consisting mainly in an increased intra-cerebral T cell recruitment and in a high reactivity of the peripheral CD4+ T lymphocytes to the Ab peptide^10,16^. The presence of a restricted subset of peripheral Ab1–42-specific CD4+ T-cells that differentiate the AD from the Lewy body dementia, evidencing their potential role in counteracting the Ab pathology^17–19^ has been described previously. In a recent study, the impact of adaptive immunity in a murine model of the AD was characterized^16^. In detail, in the APPPS1 mice, the transient depletion of the Tregs sped up the cognitive decline related to the reduced recruitment of microglia towards amyloid deposits^16^. On the other hand, the stimulation of the Tregs increased the amount of microglia associated with plaques and improved cognitive functions^7,16,20–22^.

As mentioned above, the CD4+ T cells have been subtyped with respect to their specific cytokine profiles into the T helper (Th) 1, the Th2, the Th9, the Th17, the Th22, the Tregs, and follicular helper T cells (Tfh)^14,15^. In detail, regulatory T cells have been identified as negative modulators of the immune response to antigens^14,15,23^, and have been demonstrated to exert an active regulatory role in peripheral tolerance mechanisms, whose failure leads to the development of the autoimmune disease. In this context, the breakdown of the Treg-immune control has been linked to a reduced frequency of the Tregs, to the impairment of the Treg’s suppressive function, as well as to the enhanced reactivity and resistance to the self-reactive effector, T cell regulatory machinery^24^.

The regulatory T cell compartment which includes naturally occurring and adaptively induced Tregs^13^, appears to be linked to several neurodegenerative diseases, such as to Multiple Sclerosis (MS), to the AD, to the PD, as well as to Amyotrophic Lateral Sclerosis^16,25–29^. In this context, inconsistent results have been published when the Tregs were studied in the AD. In addition, it was reported that, when the AD patients were compared to healthy subjects, frequencies decreased and increased the suppressive activity of their Tregs^30,31^, whereas, a great amount of literature is available on the role of the Treg compartment in the MS^24,27,32^. The peripheral CD4+ CD25+ Treg compartment, measured by flow-cytometry, demonstrated to be decreased^33^, or not impaired^34,35^ in the MS patients when the stable or the acute phase of the disease was analysed. However, the role of the Tregs seems to be beneficial both in the AD patients and in the MS patients, by slowing the disease’s progression and modulating the microglial response to the Ab deposition in the AD^16^, or by the migration to the inflammation site in the acute phase of the MS disease^27,32^.

It has also recently been proposed that the AD, as well as the MS, could be sustained by a similar Ab pathology^36,37^. In detail, the impaired Ab homeostasis, linked to the Ab imaging data and to the altered peripheral levels of inflammatory mediators, as well as the presence of soluble oligomers in the cerebrospinal fluid, and cognitive dysfunctions have been described as common aspects to the AD and to the MS^36–40^.

In human studies, misleading and contradictory data on Tregs have been published. These controversial results are probably due to the fact that clear guidelines for the identification and analysis of Treg do not exist. In any case, different publications have suggested that a reliable method for the whole Treg compartment identification is the flow cytometry analysis of a pattern of specific surface antigens^15,41–43^, instead of the intracellular detection of transcription factor Foxp3, which is known to be specifically expressed by the CD4+ CD25+-Tregs^44,45^. It is largely accepted that Foxp3 coordinates Treg development and functions. Mutations in the Foxp3 gene have been associated with a lethal autoimmune syndrome^46^; in any case, its exclusive intracellular expression represents a strong limitation for its application to reliable Treg studies^44,45^. Recent data have described low levels of the IL-7 receptor alpha-chain (CD127) cell surface expression on Tregs^47,48^, and an inverse correlation between the CD127surface expression levels and the intracellular expression of FoxP3 in the Tregs, and their suppressive functions^49^. In view of the above-discussed evidence, we performed the phenotypical analysis of the CD4+/CD25high/CD127low-neg Tregs, that allowed us to obtain a real picture of the analysed peripheral blood samples. Different subsets of the Tregs have been characterized by a variety of surface receptors, and/or intracellular markers and/or secreted inhibitory cytokines. The natural Tregs (CD4+/CD25 high/CD127low-neg), generated in the thymus, migrate into peripheral lymphoid organs, and then differentiate as Resting (CD45RApos/CD25dim), Secreting (CD45RAneg/CD25dim) or Activated (CD45RAneg/CD25bright) cells, thus representing the memory T cell compartments (CD45RA negative), with specific functions in the context of immune machinery^15,42,46^.

Resting Tregs represent their naïve form, before the antigen presentation process, while the Activated subset embodies the cluster which originated after their exposure to self-antigens, and the Secreting compartment has a well-described cytokine-secreting non-suppressive function^15,42^. On the other hand, we assessed their activation status through the analysis of the HLA-DR surface expression, where the HLA-DR has recently been described as a marker of the mature Treg population which acts through an early contact-dependent inhibition^50^.

Moreover, CD39 has been underlined as a functional Treg ectoenzyme, able to hydrolyse the ATP and the ADP to AMP. The antigen CD39 results expressed on a subset of regulatory effector/memory-like T cells in humans, exert its suppressive activity on activated T cells, showing anti-inflammatory functions through the production of the extracellular adenosine^1,6,46^. Of interest is that different studies have proposed the CD39+ Tregs subset as a potential marker of the inflammatory activity in the MS^32^. They have suggested that the CD4+ CD25highCD39+ T cell compartment from the MS patients in the acute phase have a suppressive function, pointing out that the T regulatory compartment is not functionally compromised in patients affected by the MS^27^. In our study, which is a pilot research, requiring a further confirmation of the results on a bigger cohort of patients, we fully analysed the Tregs (CD4+/CD25high/CD127low-neg) and their different functional subsets (Resting (CD45RApos/CD25dim), Activated (CD45RAneg/CD25bright), and Secreting (CD45RAneg/CD25dim)^15,41,42,47^ in terms of frequencies, as well as in respect to their CD39 and HLA-DR surface expression, in the AD and in the MS patients, as compared to healthy donors.

This comparative analysis allowed us to describe specific Treg profiles in the AD and in the MS, as well as similar impairments could link the AD to the MS pathophysiological mechanisms.

## Results

### Gating strategy for the treg identification and subtyping

T lymphocytes were gated on the basis of their scatter parameters (Supplemental Fig. 1A), dead cells were excluded (Supplemental Fig. 1B), then CD4+ T cells were identified (Supplemental Fig. 1C) and analysed for CD25 and CD127 surface expression: CD4+ T cells expressing high levels of CD25 (CD25high), with negative or low expression of CD127, were considered as Treg cells (Supplemental Fig. 1D). In agreement with Orrù *et al*.^42^, Tregs (CD4+/CD25high/CD127low-neg) were subtyped as Resting (CD45RApos/CD25dim), Secreting (CD45RAneg/CD25dim) and Activated (CD45RAneg/CD25bright) cells (Supplemental Fig. 1E). For all the above-reported populations of the Tregs (CD4+/CD25high/CD127low-neg, Resting, Secreting and Activated cells) the Mean Fluorescence Intensity (MFI) was obtained and the related ratio values have been calculated as reported in the Methods section. The Fluorescence Minus One (FMO) controls are also shown (Supplemental Fig. 1F–H).

### Impairment of Treg Frequencies in the AD and in the MS Patients

We used the above-described gating strategy for the Treg identification and subtyping in order to interrogate peripheral blood samples from patients affected by the AD and the MS, as well as from healthy subjects, regarding their Treg assessment (Supplemental Fig. 2A,B). Since the time of the AD and the MS patients have differed, we enrolled two cohorts of healthy subjects, matched according to age and gender with the related cohort of the patients (AD Ctrl and MS Ctrl, respectively). No statistical differences were found when the ages and gender of the AD or the MS patients were compared to them of their respective matched group of healthy volunteers (p > 0.05).

**Figure 1:**
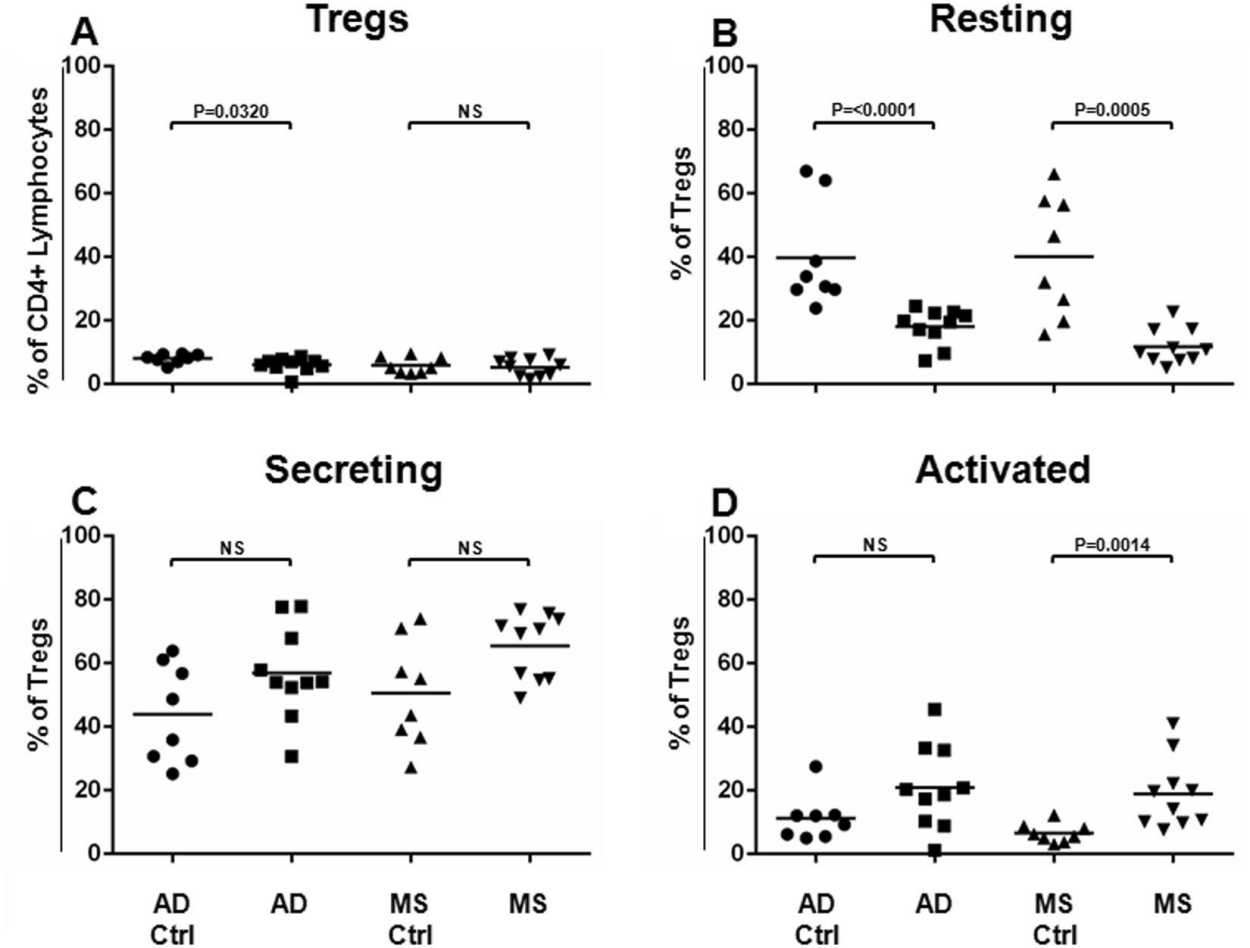
Tregs and Treg Sub-Populations in AD and MS Patients. Tregs and Treg sub-populations (Resting, Secreting and Activated cells) have been identified and analysed in AD (N = 10) and MS (N = 10) patients and compared to the related matched cohorts of healthy donors (AD Ctrl, N = 8, MS Ctrl, N = 8). Treg frequencies (% of CD4+ lymphocytes) (**A**) and each Treg aforementioned sub-population (% of Tregs) (**B**–**D**, respectively) obtained for the AD and the MS patients have been compared to those calculated for each respective matched cohort of healthy donors. Horizontal lines represent mean values. *P*-values are shown. Not significant results have been indicated as NS.

As shown in Fig. 1A, it was found that the frequency of the Tregs resulted to be significantly decreased in the AD (*P* = 0.0320), but not in the MS, as compared to the related matched healthy subject cohort. Surprisingly, when the Treg subsets were analysed, the Resting Treg compartment resulted significantly less represented in the AD (*P* < 0.0001), as well as in the MS patients (*P* = 0.0005) (Fig. 1B), in respect to the related matched healthy subject cohort (AD Ctrl or MS Ctrl, respectively).

The compartment of the Secreting Tregs slightly increased in the AD and in the MS patients, even though data did not result in being statistically significant (Fig. 1C).

The frequency of the Activated Tregs increased significantly only in the MS patients (*P* = 0.0014 Fig. 1D) when compared to the related matched healthy subject cohort.

### Treg HLA-DR Expression is Up-Regulated on the Tregs from the MS Patients

**Figure 2:**
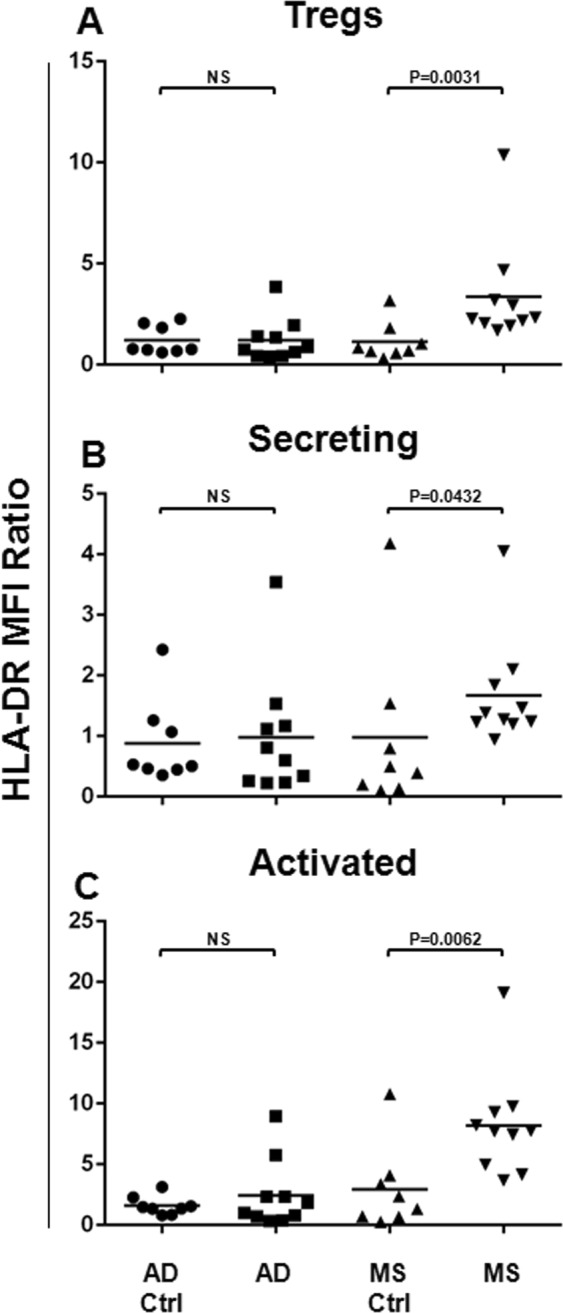
HLA-DR Surface Expression on Tregs and Treg Subsets from the AD and the MS Patients. In order to analyse the HLA-DR surface expression on Tregs and their subsets, the Mean Fluorescence Intensity Ratio (MFI-R) values for each population have been calculated. In particular, the HLA-DR expression on Tregs (**A**), and on Secreting (**B**) and Activated (**C**) subsets has been compared between AD (N = 10) or MS (N = 10) patients and the respective matched healthy subject cohort (AD Ctrl, N = 8, MS Ctrl, N = 8). Horizontal lines represent mean values. *P*-values are shown. Not significant results have been indicated as NS.

Since the HLA-DR has been indicated as a Treg maturation and activation marker^50^, we aimed to analyse its expression on the total Tregs, as well as on each Treg subset, both in the AD and in the MS patients. For this reason, as shown in Fig. 2, its surface expression was assessed for the Tregs (Fig. 2A), as well as for Secreting (Fig. 2B) and Activated (Fig. 2C) cells. The Mean Fluorescence Intensity (MFI) ratio values were compared between the AD or the MS patients and the respective matched healthy subject cohort. Statistical analysis shows that the HLA-DR resulted up-regulated on total Tregs (*P* = 0.0031) from the MS patients, with respect to its related matched healthy subject cohort (Fig. 2A). In particular, the HLA-DR expression resulted to be more expressed on Secreting (*P* = 0.0432, Fig. 2B), as well as on Activated (*P* = 0.0062, Fig. 2C) Tregs. In Alzheimer Disease patients, the surface expression of the HLA-DR on Tregs and their subsets did not result impaired (Fig. 2A–C).

### Treg CD39 Expression in the AD and in the MS Patients

**Figure 3:**
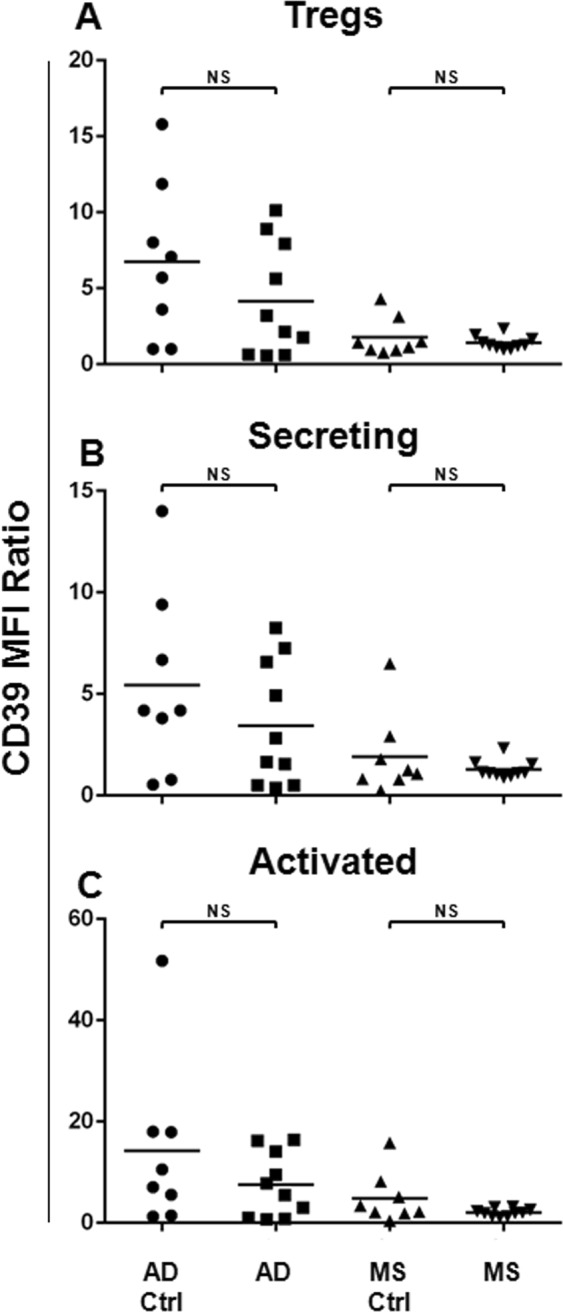
CD39 Surface Expression on Tregs and Treg Subsets from the AD and the MS Patients. In order to analyse the CD39 surface expression on Tregs and their subsets, Mean Fluorescence Intensity Ratio (MFI-R) values for each population have been calculated. In particular, the CD39 expression levels on Tregs (**A**), Secreting (**B**) and Activated (**C**) subsets have been compared between the AD (N = 10) or the MS (N = 10) patients and the respective matched healthy subject cohorts (AD Ctrl, N = 8, MS Ctrl, N = 8). Horizontal lines represent mean values. *P*-values are shown. Not significant results have been indicated as NS.

It has been demonstrated that CD39 represents a functional Treg marker, mediating both the suppressive and anti-inflammatory functions^32,46^. Therefore, we assessed the CD39 surface expression on Tregs and their subsets, comparing its expression between the AD or the MS patients and the respective matched healthy subject cohort (Fig. 3). Data demonstrated that in the MS and in the AD patients, no impairment in terms of the CD39 surface expression on the Tregs and their subsets was observed.

## Discussion

In the present study, the assessment of the Tregs in the AD and in the MS patients and possible phenotype similarities in their Treg compartments was described. Regulatory T cells were analysed in a quantitative fashion, and their possible contribution to the AD and the MS physiopathology has been discussed. In particular, the relationships among numbers and phenotypes of regulatory CD4+ T cells, the adaptive immunity and the related neurodegeneration have been addressed.

We analysed the Treg compartment by Polychromatic Flow Cytometry, applying a panel that allows the identification of the Tregs by an existing established phenotype (CD4+/CD25high/CD127low-neg)^15,42,43,47^ that instead of the common panels for the Treg characterization (mainly based on the intracellular FoxP3 detection), allows to carry out of the analysis on non-manipulated samples non-fixed and non-permeabilized cells. This guaranteed the possibility to obtain a more reliable picture of the peripheral blood situation.

Recent literature has described the active regulatory role of the Tregs in peripheral tolerance mechanisms linked to the development of the autoimmune disease. Regulatory T cells could fail in the peripheral immune surveillance functions through different mechanisms, such as the reduced frequency of the Tregs, or impairment of the Treg suppressive function or of an enhanced reactivity and resistance to the self-reactive effector T cell regulatory machinery^24^.

Based on this evidence we applied the innovative Polychromatic Flow Cytometry panel described above, in order to study the immune-phenotype of the Tregs in the AD and in the MS diseases. Interestingly, we found that the percentage of Tregs significantly decreased in the AD, as compared to a matched cohort of healthy subjects. These results suggest impaired systemic immunosuppression in the AD physiopathology, which recalls the possibility that the decrease of Tregs could contribute to the well-described failure of the Treg immune-surveillance in the AD.

We also analysed the Treg profile subtyped into Resting, Activated and Secreting subsets, applying the above-mentioned panel^15,42,43,47,48^ in the AD, in the MS and in related matched healthy control cohorts. Surprisingly, we observed a decrease of Resting Treg frequencies both in the AD and in the MS patients, with respect to the related healthy subject cohorts. Resting Treg cells differentiate as activated Tregs after the antigen exposition. Their decrease, both in the AD and in the MS patients, could describe an impairment of the immune reserve in both mentioned pathologies, possibly linked to a further breakdown of the Treg-mediated peripheral tolerance barriers.

In order to assess the activation status of the Treg in the AD and in the MS, we also evaluated the HLA-DR surface expression, which has been referred to as a Treg differentiation marker^25,50^. In our setting, the HLA-DR surface expression was up-regulated on the total Tregs as well as on Secreting and Activated Treg subsets from the MS patients, with respect to the related cohort of healthy subjects. Instead, in AD patients, the HLA-DR surface expression on the Tregs and on their subsets did not result impaired. These data describe the presence of a more abundant terminally differentiated Treg sub-population in the MS, but not in the AD. The abundance of those cells can be considered as an immune activation index, therefore reflecting the acute *versus* the chronic immune activation status in both the MS and in the AD.

In this context, we also analysed the CD39 surface expression levels, which is reported to mediate both suppressive and anti-inflammatory activities. Recent studies have suggested that the catalytic inactivation and the conversion of the extracellular ATP and ADP to the AMP, induced by CD39, needs to be considered as an anti-inflammatory mechanism by which the Tregs mediate the immune suppression in human autoimmune diseases, such as in the MS^32,46^.

Here, we observed that the CD39 surface expression did not result impaired in the AD or in MS patients.

The reported characterization of the T regulatory compartment in the AD and in the MS underlines interesting similarities and differences in terms of Treg profiles. A parallel and interesting decrease of the resting Treg subset in the AD, as well as in the MS could be associated to a common impairment of the immune competences, possibly representing a failure mechanism in the Treg-mediated peripheral tolerance barrier efficacy, which could characterize the above-mentioned pathologies.

Altogether, these data allowed us to propose the reduction of total Treg frequencies, as well as the decrease of the Treg Resting fraction as a possible mechanism contributing to the failure of the Treg-mediated immunological tolerance in AD. The major weakness of the present study is its pilot nature, given that it relies on a small sample size, therefore providing preliminary data and their validation is outside of its scope, needing to be validated in a larger cohort of patients.

The implication of autoimmune mechanisms in the AD physiopathology could open new possible prognostic applications linking the Tregs profile to the AD grading. In addition, the amplification of Tregs with a low-dose IL-2 treatment has been shown to be well tolerated in different clinical pathological conditions^51^ and in AD murine models^16^, supporting possible immunotherapeutic prospective based on the modulation of Tregs in the AD.

## Methods

The present study was approved by the local Ethical Committees (Protocol No. 176, of the Ethics Committee of University “G.d’Annunzio”, Chieti-Pescara and of the ASL n.2 Lanciano-Vasto-Chieti, Italy), and was carried out according to the Declaration of Helsinki and subsequent revisions (World Medical Association Declaration of Helsinki, 1997). All participants signed a written informed consent after having been informed about the procedures of the study.

### Patients

A total of 36 subjects were enrolled. Ten (10) patients with a definite clinical MS diagnosis (male = 3, female = 7; mean age = 39), in the acute phase of the disease, were recruited by the Unit of Neurology, IRCCS Neuromed Institute (Italy) and the MS Relapsing-Remitting (RR) condition was established according to the relative state of the art diagnostic criteria^52^; 10 middle stage Alzheimer’s Disease patients (male = 3, female = 7; mean age = 70), 8 AD matched healthy subjects (AD Ctrl; male = 3, female = 5; mean age = 63) and 8 MS matched healthy volunteers (MS Ctrl; male = 3, female = 5; mean age = 36) were randomly selected by the Memory Clinic of the Department of Neuroscience and Imaging (University “G. D’Annunzio”, Chieti-Pescara, Italy). The diagnosis of probable Alzheimer’s Disease was based on the current criteria of the National Institute of Neurological Disorders and Stroke-Alzheimer’s Disease and Related Disorders Association (NINDS-ADRDA)^53^ all cardinal features (Part I) and at least 2 supportive features (Parts II and III) were diagnosed. All AD patients underwent psychometric examinations and Magnetic Resonance Imaging (MRI). The following exclusion criteria were applied: other systemic comorbidities (immune, neoplasm, respiratory, renal, liver or cardiac failures), recurrent urinary or pulmonary infections, and pregnancy. None of the patients was treated with steroids or immunosuppressive agents. Demographic features of the above-mentioned patients are summarized in Table 1.

**Table 1:** Demographic Characteristics of Patients.

		AD Ctrl	AD	MS Ctrl	MS
**Number of observations**		8	10	8	10
Age	Mean	63	70	36	39
	Std. Deviation	9	10	13	13
	Minimum	54	45	19	17
	Maximum	79	79	53	52
Gender	Female	5	7	5	7
	Male	3	3	3	3

### Flow cytometry

From each enrolled subject, Peripheral Blood (PB) was drawn in EDTA (2 mg/mL) tubes (BD K2E EDTA, Becton Dickinson Biosciences - BD, San Jose, CA, USA), and processed within 2 hours from bleeding. For each sample, 100 μl of PB was processed by a common flow cytometry lyse and wash method. Briefly, PB samples were stained using a panel of lyophilized reagents which are detailed in Supplemental Table 1. After 30 min of staining (4 °C in the dark), samples underwent an erythrocyte-lyse step, with 1 mL of 1X Pharm Lysing solution (BD, Biosciences), for 15 min at room temperature, with gentle agitation, according to the manufacturer’s instructions. Samples were then centrifuged (400 g, 10 min, room temperature) and washed by adding 2 ml of 1X PBS and 1.5 × 10^5^ events/sample were acquired by Flow Cytometry (FACSCanto II, three lasers, eight colour configuration, BD Biosciences). The threshold was placed on the Forward Scatter (FSC) channel. Tregs and their subsets were identified as reported^15,42,43,47^. Data were analysed by using FACDiva v6.1.3 software (BD Biosciences). To ensure the correct identification of negative and positive populations, cells were plotted using the dot-plot bi-exponential display^19,54,55^. Instrument performances, data reproducibility, and fluorescence calibrations were sustained and checked by the Cytometer Setup & Tracking Module and further validated by the acquisition of Spherotech 8-peck Rainbow Beads (BD). In order to evaluate non-specific fluorescence, FMO controls were used^56,57^. The compensation was assessed using CompBeads (BD) and single stained fluorescent samples. Given that the MFI can differ among different experiments, it needs to be standardized to allow comparisons among different subjects. Here, the MFI values (i.e. HLA-DR and CD39) were standardized by calculating related MFI ratios, obtained by dividing each MFI by the related MFI of the CD4- Lymphocytes, used as an internal reference^19,58^. Data were analysed using FACSDiva v 6.1.3 (BD), FACSuite v 1.0.5 (BD) and FlowJo v 8.8.6 (TreeStar, Ashland, OR, USA) software.

### Statistical analysis

Statistics of flow cytometry data (referred to the frequencies of Tregs, their subsets, and related MFI ratios) were performed using the XLSTAT 2014 (Addinsoft, Paris, France) and the GraphPad Prism 6 (GraphPad Software, La Jolla, CA, USA) software. No assumptions of normality were formulated and no values were excluded, hence the non-parametric Mann Whitney *U* test, which was used for comparisons. Gender homogeneity was assessed by Fisher’s exact test. Statistical significance was accepted for P < 0.05 (two-tailed).

## Supplementary information

Supplemental Figure 1, Supplemental Figure 2 and Supplemental Table 1
