# Supplementary material for: The Characterization of Regulatory T-Cell Profiles in Alzheimer’s Disease and Multiple Sclerosis
Source: Sci Rep. 2019 Jun 19;9:8788. doi: 10.1038/s41598-019-45433-3 (PMC6584558; doi:10.1038/s41598-019-45433-3)
Supplement: Supplementary file 1 — Supplemental Figure 1, Supplemental Figure 2 and Supplemental Table 1 [file 41598_2019_45433_MOESM1_ESM.pdf]

The Characterization of Regulatory T-Cell Profiles in Alzheimer's Disease and Multiple Sclerosis

Fausta Ciccocioppo[1,3], Paola Lanuti[1,3], Laura Pierdomenico[1,3], Pasquale Simeone[1,3], Giuseppina Bologna[1,3], Eva Ercolino[1,3], Fabio Buttari[5], Roberta Fantozzi[5], Astrid Thomas[2,3], Marco Onofrj[2,3], Diego Centonze[4,5,*], Sebastiano Miscia[1,3] and Marco Marchisio[1,3]

[1]Department of Medicine and Aging Sciences, University "G. D'Annunzio" Chieti-Pescara, Italy
[2]Department of Neuroscience, Imaging and Clinical Sciences, University "G. D'Annunzio" Chieti-Pescara, Italy
[3]Center on Aging Science and Translational Medicine (Ce.S.I.-Me.T.), University "G. D'Annunzio" Chieti-Pescara, Italy
[4]Multiple Sclerosis Clinical and Research Unit, Department of Systems Medicine, Tor Vergata University, Rome, Italy
[5]IRCCS Istituto Neurologico Mediterraneo (INM) Neuromed, Neurology and Neurorehabilitation Units, Pozzilli, (IS), Italy

F.C. and P.L. The Authors contributed equally in the study.
S.M. and M.M. The Authors share Senior Authorship.

***Corresponding Author:** Diego Centonze MD, PhD. Multiple Sclerosis Clinical and Research Unit, Department of Systems Medicine, Tor Vergata University, Via Montpellier 1, 00133 Rome, Italy. Telephone: +39 06 7259 6010; Fax: +39 06 7259 6006. E-mail: centonze@uniroma2.it

Supplemental Figure 1

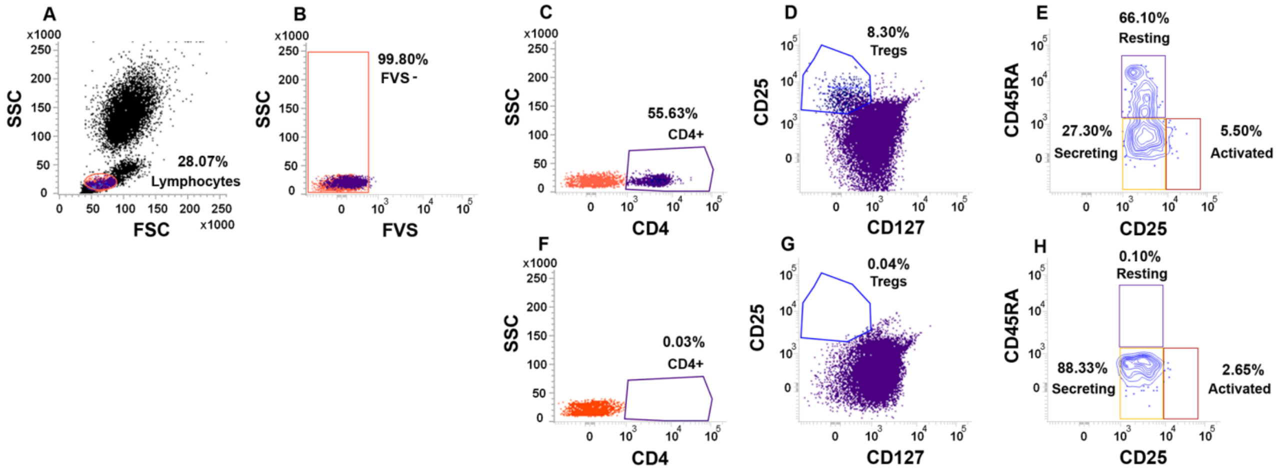

Gating strategy for Treg identification and sub-typing. Lymphocytes were gated on scatter parameters SSC-A and FSC-A (A). Live cells have been identified on the basis of their negative staining to the Fixable Viability Stain (FVS) (B). Tregs were identified as CD4+ events (C), CD25+ and CD127low/- (D). Tregs were than subtyped as Resting (CD45RA+CD25+), Activated (CD45RA-CD25bright) or Secreting (CD45RA-CD25+) (E). FMO for CD4 (F) CD25 (G), and CD45RA (H) are shown. The gating strategy here described is representative of all analysed samples.

Supplemental Figure 2

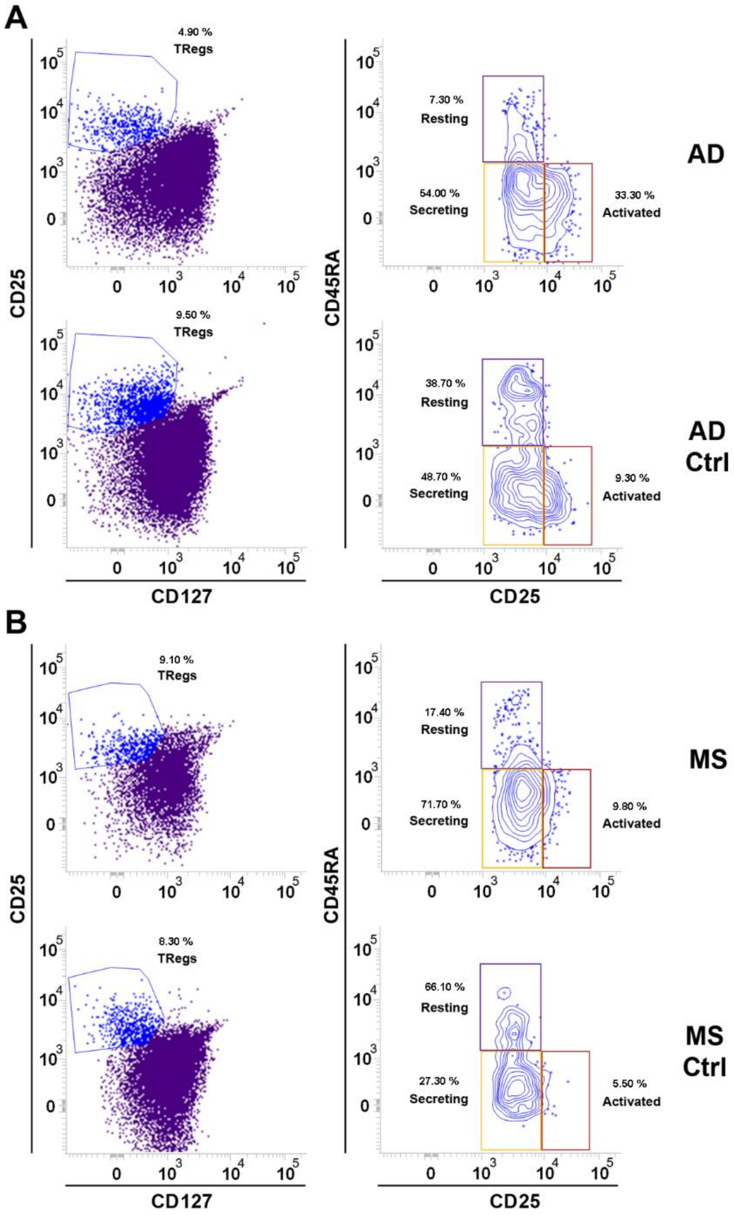

Tregs and Treg subsets in AD and MS patients. Treg identification and subtyping gating strategy are shown for AD and AD Ctrl subjects (A), as well as for MS and MS Ctrl subjects (B). Data are representative of the analysed dataset.

Supplemental Table 1. List of flow cytometry specificities and reagents.

Detection	Fluorochrome	Vendor	Ab Clone	Catalogue Number
BD Horizon™ Fixable Viability Stain (FVS) 450	V450 (equivalent)	BD Biosciences	-	562247
CD45RA	FITC	BD Biosciences	L48	624637*
CD25	PE	BD Biosciences	2A3	624637*
CD127	PerCP-Cy™5.5	BD Biosciences	HIL-7R-M21	624637*
HLA-DR	PE-Cy™7	BD Biosciences	L243	624637*
CD39	APC	BD Biosciences	TU66	624637*
CD4	APC-H7	BD Biosciences	SK3	624637*

*Catalogue number of the lyophilized combination. Keys: Fluorescein Isothiocyanate (FITC); R-phycoerythrin; (PE); Peridinin-chlorophyll protein- Cyanine 5.5 (PerCP-Cy 5.5); Allophycocyanin (APC); APC-Hilite®7 (APC-H7). Becton Dickinson (BD) Biosciences (San Jose, CA, USA).
